# Anorectal Malformations (Part 1)

**Published:** 2015-01-10

**Authors:** Sushmita Bhatnagar

**Affiliations:** Department of Pediatric Surgery, B.J.Wadia Hospital for Children, Mumbai

 (This section is meant for residents to check their understanding regarding a particular topic)

## Questions

1. What are the various types of anorectal malformations (ARM)?
2. What is the pathophysiology of ARM?
3. What are the clinical features of newborn with anorectal malformation?
4. What are the radiological investigations required for diagnostic evaluation?
5. What are its associated anomalies?

## Answers

**Answer 1**

Anorectal malformations comprise a wide spectrum of anomalies of the anorectal system, urogenital system, sacral spine and perineal musculature. The extent of anomalies in these four components decides the type of anorectal malformation.

Gender variations in the type of malformations must also be clearly defined before primary workup and management plan is drawn.

Based on the anatomy, various classifications have been proposed to define the pathology of these anorectal anomalies. The earliest classification dates back to 1953 when Gross proposed a simple differentiation based on the levator muscle (Fig.1), i.e. supralevator – for those above the levator ani or infralevator anomalies, for those below the levator ani. [1]

**Figure:**
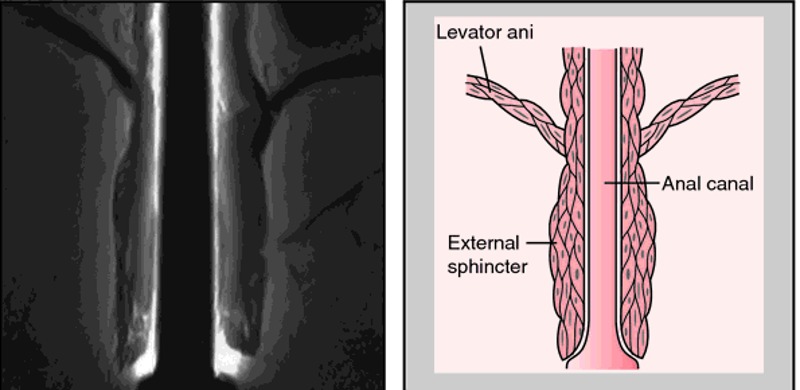
Figure 1: Diagrammatic representation of levator ani.

With advancement in the understanding of the pathology of the malformations, a need was felt to define these lesions more appropriately. During a meeting to celebrate the centenary of the Melbourne Royal Children’s Hospital, a new International classification was proposed in 1970 [2,3] as shown in Table 1. This classification utilized the concept of levator ani wherein anomalies above the levator were termed as high and those below were termed as low anomalies, but it also introduced intermediate anomalies which were known as translevator anomalies. 

**Figure:**
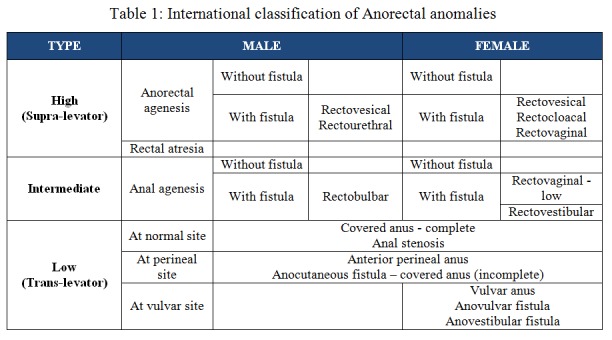
Table 1: International classification of Anorectal anomalies

In 1984, during a conference on Ano-rectal malformations organized by Prof. D.Stephens and Prof. D.Smith Wingspread, Wisconsin, another classification was proposed. [4,5] This classification also included the special groups in cloacal and rare malformations as shown in Table 2. 

**Figure:**
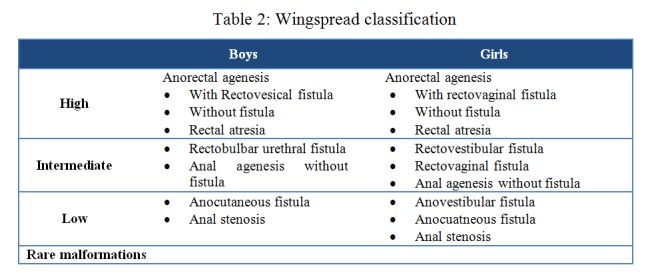
Table 2: Wingspread classification

By the early 1980’s, several other rare anomalies such as perineal groove, H type of anorectal anomalies, pouch colon, rectal ectasia, rectal atresia, etc. were introduced and documented which were not included in the Wingspread’s classification. Also, neither the surgical procedures nor the protocols for assessing post-operative outcome were standardized. Thus, in 1995 Pena introduced a disparate classification system as shown in Table 3. 

**Figure:**
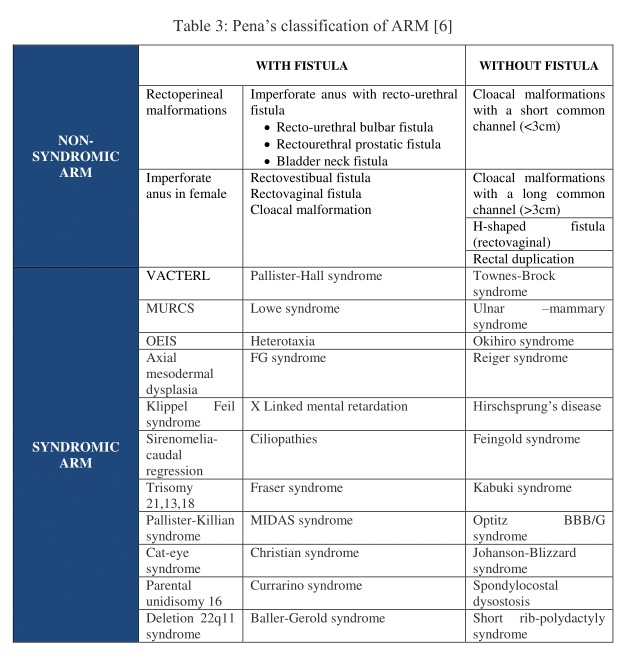
Table 3: Pena’s classification of ARM [6]

May 2005, 21 years after the Wingsrpead classification saw the Krickenbeck meeting organized by Professor Alex Holschneider from Cologne, Germany.[7] The goal of the meeting was to develop international criteria for treatment and develop a uniform scoring system for comparable follow-ups. The Pena’s classification was modified as per the type of fistula and included rare variants as shown in table 4.

**Figure:**
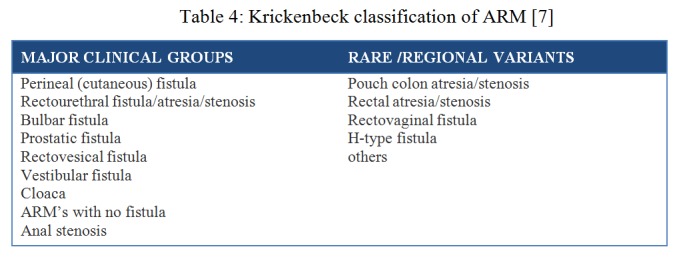
Table 4: Krickenbeck classification of ARM [7]

**Answer 2 **

Embryologically, interference in the development of anorectal and genitourinary organs at various stages upto 7 to 8 weeks of gestation gives rise to a range of anomalies from mild to severe abnormalities involving even the musculoskeletal system of the hindgut. Continued communication between the urogenital tract and rectal portions of the cloacal plate causes rectourethral fistulas or rectovestibular fistulas. [8]

Till date, the accurate embryologic defect causing anorectal malformations still remains undetermined. Nevertheless what is known is that defects in formation or shape of cloacal membrane formation and subsequent breakdown into urogenital and anal openings, which occurs by 8 weeks of gestation, are responsible for the numerous abnormalities of the anorectum. The incorporation of Mullerian ducts, which are formed later, into the anomalous development is also not clear. The pelvic floor as well as the external anal sphincter, derived from exterior mesoderm, is usually present but has varying degrees of anomalies which range from normal musculature to absent muscle complex. The higher the rectal pouch, more are the chances of mal-development of the pelvic floor.

With recent researches in the pathogenesis of anorectal malformations, the previous theories have been discarded. While in the past, defects in lateral fusion were thought to be causative, there is evidence from animal models and from detailed study of human fetuses with major anomalies that a deficiency in the dorsal component of the cloacal membrane and the adjacent dorsal cloaca is causative. A subsequent malfunction of the primitive streak and tail bud in the early development phase around 3-4 weeks has been proposed (yet to be clearly defined) as causation for associated anomalies of the pelvic floor.

The histological analysis of specimens from human fetuses with non-viable malformations revealed the following findings : [9]

1. Primarily, the maldevelopment affects the anal canal and rectum is secondarily affected.
2. There is ventral displacement of anal canal which opens either on the perineum or forms a fistula to urogenital tract.
3. Those malformations in which a fistula is not demonstrated, a rudimentary partly regressed connection is found on histology.
4. In those with fistula from rectum to urogenital structures, there is a gradual transition of the anal mucosa to urogenital mucosa.
5. In proximal fistulae, the development of trigone of bladder, the upper urethra and the urethral sphincter is also abnormal in males whereas in females, vaginal development is inappropriate causing a urogenital sinus caudal to mesonephric ducts (as seen in persistent cloaca).
6. With deficient anal canal, the striated muscles of the perineum often have abnormal configuration. The longitudinal fibers of external anal sphincter are concentrated medially, the bulbospongiosus muscle is displaced medially in high lesions and the puborectalis sling, the external urethral sphincter and ischiocavernous muscles are variably affected depending on the severity of the lesion.
7. The likelihood of associated abnormalities in the development of pelvis, perineum, bladder, ureters, phallus etc. were proportional to the length of agenesis as measured from the actual anal site.

**Answer 3 **

A thorough clinical assessment (substantiated with radiological assessment when needed) is essential for accurately classifying the malformation as the choice of surgical treatment is largely dependent on the extent of the anomaly. 

The important aspects in history and clinical examination are listed in tabular form as shown in Table 5.

**Figure:**
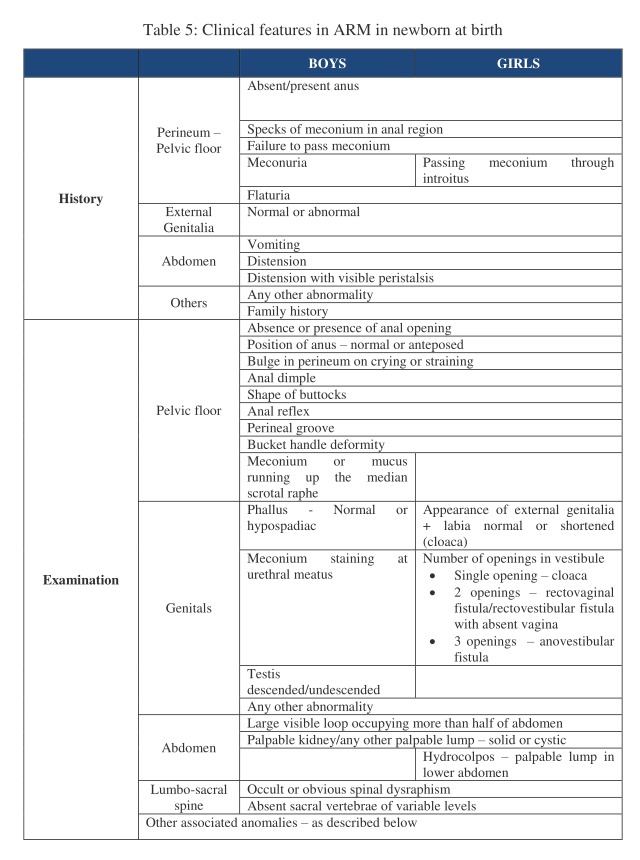
Table 5: Clinical features in ARM in newborn at birth

**Answer 4 **

Assessment of the type of anomaly often needs radiological assistance in the form of x-rays or ultrasonography. Few associated anomalies also need to be investigated at the time of birth, especially the genitourinary and cardiac lesions. 

The timing and method of radiological investigations are tabulated as in Table 6 as follows.

**Figure:**
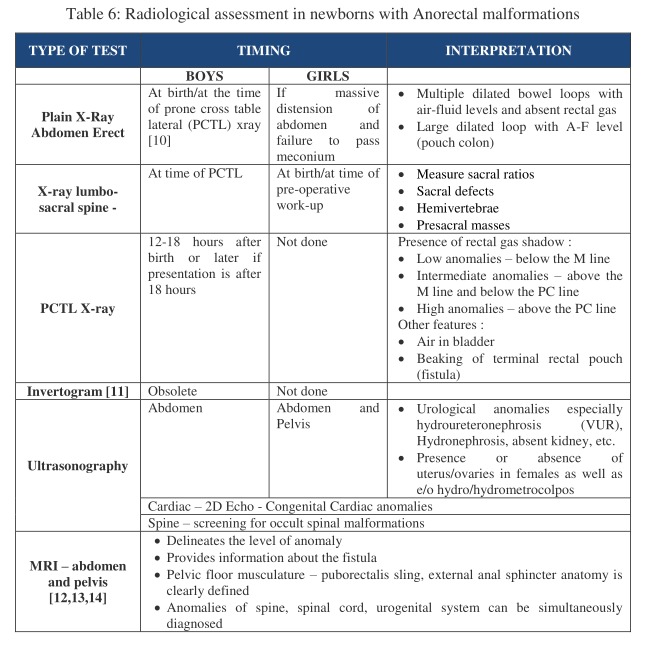
Table 6: Radiological assessment in newborns with Anorectal malformations

**Answer 5 **

Anorectal malformations present with a high incidence of associated anomalies. The anomalies are presented in a tabular form in the decreasing order of frequency as shown in table 7.

**Figure:**
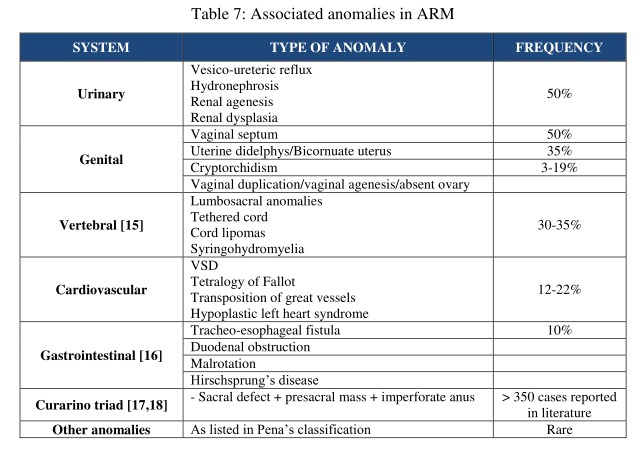
Table 7: Associated anomalies in ARM

## Footnotes

**Source of Support:** Nil

**Conflict of Interest:** The author is editor of the journal. The manuscript is independently handled by other editors and she is not involved in decision making about the manuscript.

